# Predicting Long-Term Ventricular Arrhythmia Risk in Children with Acute Lymphoblastic Leukemia Using Normal Values of Ventricular Repolarization Markers Established from Japanese Cohort Study

**DOI:** 10.3390/jcm12144723

**Published:** 2023-07-17

**Authors:** Masahiro Takeguchi, Satoshi Kusumoto, Kazuhito Sekiguchi, Souichi Suenobu, Kenji Ihara

**Affiliations:** Department of Pediatrics, Oita University Faculty of Medicine, 1-1 Idaigaoka, Hasama, Yufu 879-5593, Oita, Japan; civicvti@oita-u.ac.jp (M.T.); k.sato.o1635@gmail.com (S.K.); sekiguch@oita-u.ac.jp (K.S.); suenobu@oita-u.ac.jp (S.S.)

**Keywords:** acute lymphoblastic leukemia, anthracyclines, ventricular repolarization, Tpeak-end interval, Tpeak-end/QT ratio

## Abstract

Background: Cardiac complications due to anthracycline treatment may become evident several years after chemotherapy and are recognized as a serious cause of morbidity and mortality in cancer patients or childhood cancer survivors. Objectives: We analyzed ventricular repolarization parameters in electrocardiography for pediatric acute lymphoblastic leukemia patients during chemotherapy and in long-term follow-up. To establish the reference values of ventricular repolarization parameters in children, we retrospectively summarized the Tpe interval, QT interval, QTc interval, and Tpe/QT ratio in healthy Japanese children. Methods: Electrocardiography data recorded from students in 1st and 7th grades were randomly selected from a database maintained by the school-based screening system in the Oita city cohort, Japan. Subsequently, chronological data of the Tpe/QT ratio in 17 pediatric patients with acute lymphoblastic leukemia were analyzed over time. Results: The mean ± standard deviation of the Tpe interval in 1st and 7th graders was 70 ± 7 and 78 ± 17 ms, respectively, while the mean ± standard deviation of the Tpe/QT ratio was 0.21 ± 0.02 and 0.22 ± 0.02 ms, respectively. During the intensive phase of treatment, the Tpe/QT ratios of 3 high-risk patients among the 17 patients with acute lymphoblastic leukemia exceeded the upper limit. Conclusion: The Tpe/QT ratio has a potential clinical application in predicting the risk of long-term ventricular arrhythmia of cancer patients or childhood cancer survivors from childhood to adulthood.

## 1. Introduction

Anthracyclines represent a widely applied antineoplastic agent and are recognized as a key drug for combination chemotherapy for acute lymphoblastic leukemia (ALL) in children [1,2]. However, the development of cardiac complications due to anthracycline treatment in a dose-dependent manner may become evident several years after chemotherapy [3]. Some patients may even develop malignant arrhythmias, such as tip torsion ventricular tachycardia, hence the serious cardiac complications, especially occurrence of ventricular arrhythmia (VA), are recognized as a significant cause of morbidity and mortality in cancer patients or childhood cancer survivors [3,4].

In addition to the chemotherapy for ALL, life-threatening VAs are serious problems for pediatric patients with long QT syndrome (LQTS), hypertrophic cardiomyopathy, sequelae of Kawasaki disease [5]. Traditional electrocardiography (ECG) markers that are used to predict the risk of VA have mainly focused on abnormal ventricular repolarization (VR), based on the parameters of the correct QT (QTc) interval or QT dispersion (QT-d) [6]. The QT-d, defined as the maximum difference of the QT interval between two leads in 12-lead ECG, had been proposed as a surface ECG marker of vulnerability to VA and a potential predictor of mortality, but the clinical application of QT-d has been limited, both because of probable flawed assumptions [7,8,9] and large overlap between healthy subjects and patients with cardiac disease [10].

The Tpeak–end (Tpe) interval on ECG represents only repolarization in the ventricle, although the QTc interval represents both depolarization and repolarization in the ventricle [11]. The prolongation of the Tpe interval is reported to be associated with a predisposition to life-threatening VA in LQTS, and Brugada syndrome (BS) [5,6]. From a large-scale epidemiological study, the U-shaped association between a prolonged Tpe interval and increased risk of all-cause or cardiovascular mortality and atrial fibrillation with heart failure in elderly patients has been reported [12]. The dispersion of Tpe intervals (Tpe-d) has been used to predict malignant VA and sudden cardiac death in patients with myocardial infarction [6] and BS [13], but this measure likely suffers from similarly flawed assumptions as QT dispersion [7,8,9]. Tpe-d has also been shown to be effective in predicting malignant ventricular fibrillation in BS [13]. Since the Tpe interval is considerably affected by heart rate and varies among races, with significant inter-individual variability, the Tpe/QT ratio has been clinically applied to predict the risk of arrhythmia in LQTS, BS, acute myocardial infarction [14,15], and pediatric sepsis [16]. The clinical application of the Tpe interval and Tpe/QT ratio as ECG markers for VR has been limited in children [17,18,19], partly because the reference values for these parameters—particularly those classified by age during childhood—have not been established.

The aim of this study was to evaluate the ECG markers for VR in childhood ALL, which may cause life-threatening cardiac complications. For this purpose, we first attempted to establish the reference values of the Tpe interval and Tpe/QT ratio in healthy Japanese children.

## 2. Methods

### 2.1. Study Population

#### 2.1.1. ALL Patients

From April 2002 to November 2011, 22 children were newly diagnosed with B-precursor ALL in Oita University Hospital and were treated according to the Japan Association of Childhood Leukemia Study (JACLS) ALL-02 protocol. The outline of the JACLS ALL-02 protocol was reported previously [1]. The ECG evaluation was performed before treatment, during the intensive phase of treatment, as needed, and once every 1–5 years after the end of treatment, depending on anthracycline treatment. In this study, we excluded 5 patients with underlying disease (21 trisomy or renal disease) or with a lack of appropriate ECG records. Ultimately, 17 patients were included in the study (11 boys and 6 girls) (Table 1). The age at onset ranged from 1 to 11 years (median age, 4 years). The follow-up period was 2–15 years (median, 11 years). According to the ALL-02 risk criteria, patients were classified and treated with the protocols for standard-risk (SR; *n* = 5), high-risk (HR; *n* = 9), extremely-high-risk (ER; *n* = 2), and Philadelphia chromosome-positive (Ph; *n* = 1) ALL. Two patients relapsed after the initial chemotherapy (1 SR patient and 1 ER patient). All children were treated with variable doses of anthracycline according to the protocols. The cumulative dose of anthracycline ranged from 54 to 246 mg/m^2^ (as doxorubicin equivalent). The children whose leukemia relapsed or who underwent hematopoietic cell transplantation received additional anticancer drugs, including anthracycline.

#### 2.1.2. Standard Population for Children

**Figure 1:**
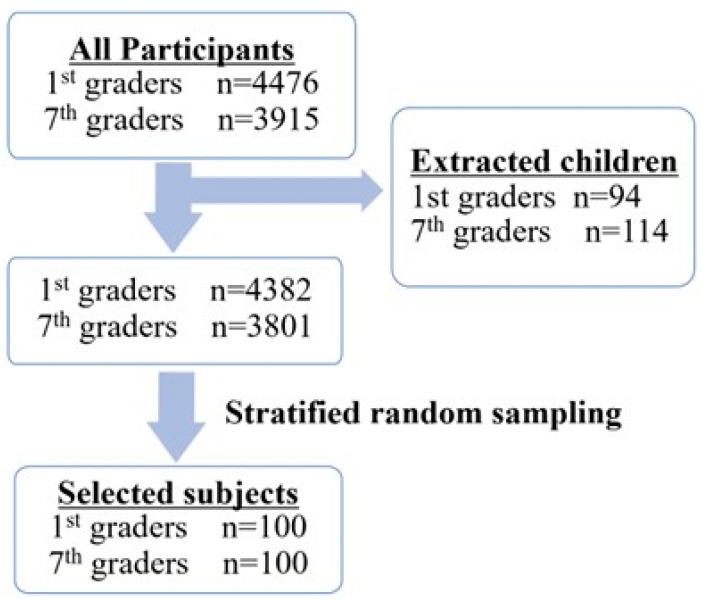
Flowchart of enrollment of school-aged children in the present study.

The healthy pediatric subjects included in the present study were selected from a database of participants in a school-based screening system in Oita city in Japan in 2017. The population included 4476 1st graders (6–7 years of age, 2274 boys and 2202 girls), and 3915 7th graders (12–13 years of age, 2029 boys and 1886 girls). The school-based screening program for cardiovascular diseases in Oita consisted of three steps. All students underwent initial screening using 12-lead resting ECG and a questionnaire sheet, which was completed by their parents. The secondary test was performed for children extracted based on the Guideline for Selecting Candidates for Secondary Screening of Heart Disease in Schools (Japanese Society of Pediatric Cardiology and Cardiac Surgery 2006) [20], such as the presence of a family history of arrhythmia, sudden death, congenital heart disease, or a personal history of heart disease or other special diseases identified by the questionnaires. As a result, 94 1st graders and 114 7th graders underwent the second examination; these students were excluded from the analysis to eliminate the possibility of underlying cardiovascular disease. One hundred 1st graders and 100 7th graders were extracted by stratified random sampling from 4382 1st graders and 3801 7th graders. The male/female ratio was 49/51 for 1st graders and 56/44 for 7th graders (Figure 1, Table S1).

The electrocardiograms included in the present study were selected from the ECG database of students who participated in a school-based screening system in Oita, Japan. The participants included 100 1st graders and 100 7th graders who were selected by stratified random sampling.

### 2.2. Ethics

The study of the school-based screening program was approved by the Ethics Committee of the Oita University Faculty of Medicine (reference No. 1553), and the participants were informed about research opportunities using opt-out approach. The study of JACLS ALL-02 was approved by the institutional review board (IRB) of Oita University Hospital (IRB No. B02-031), and written informed consent was obtained from the patients and/or their guardians.

### 2.3. ECG in the Present Study

Electrocardiograms obtained in the school-based screening program were recorded using a portable PC-based system (Fukuda Denshi, Tokyo, Japan) at a speed of 25 mm/s, a sampling rate of 500 Hz, and a bandwidth of 0.5–35 Hz by authorized technicians with supervisors at each recording. Electrocardiograms were processed using a 12-lead ECG analysis program (S2 Version; Fukuda Denshi) based on an international standard from the International Electrotechnical Commission (IEC 60601-2-25) [21]. Electrocardiograms of patients with ALL were recorded using a Cardiofax (Nihon Kohden) with a 150 Hz low-pass filter.

### 2.4. Measurement and Calculation of Ventricular Repolarization Markers

Two observers manually measured the Tpe interval, QT interval, and RR interval from ECG records from 100 children in each grade. For the evaluation of inter-observer variability, the Tpe intervals from 30 records that were randomly chosen by the two observers were statistically evaluated.

The Tpe interval, QT interval, QTc interval, and Tpe/QT ratio were measured and/or calculated as follows: the Tpe interval was measured in each precordial lead from the peak to the end of the T-wave. The end of the T-wave was defined as the isoelectric line intersecting a tangential line drawn at the maximal downslope of the positive T-wave. The Tpe interval of V5 was adopted as a representative value [13]. When the end of the T-wave was ambiguous because of low-amplitude (<0.1 mM), the data from these leads were excluded from the analysis. The QT interval of 3 consecutive beats was measured from the start of the Q wave to the end of the T-wave in lead V5. Bifid T-waves, but not U waves, were included in the QT interval. When the notch was recorded in more than 3 leads and the notch appeared at the same timing, the T-wave was defined as the bifid T-wave [22,23]. The QT/RR^1/3^ values from each of 3 consecutive beats were calculated using Fridericia’s formula and the mean values of the 3 consecutive waves represented the QTc interval. The Tpe/QT ratio from the lead V5 was defined as the representative value [24].

### 2.5. Statistical Analysis

The data are presented as the mean ± standard deviation (SD). All statistical analyses were performed with EZR (Saitama Medical Center, Jichi Medical University, Saitama, Japan), which is a graphical user interface for R (The R Foundation for Statistical Computing, Vienna, Austria) [25]. The Shapiro–Wilk test was used to determine whether the measurements followed a normal distribution. The unpaired Student’s *t*-test was applied to evaluate differences between two groups. *p* values of <0.05 were considered to indicate statistical significance.

## 3. Results

### 3.1. Repolarization Parameters of the Control Pediatric Subjects

**Figure 2:**
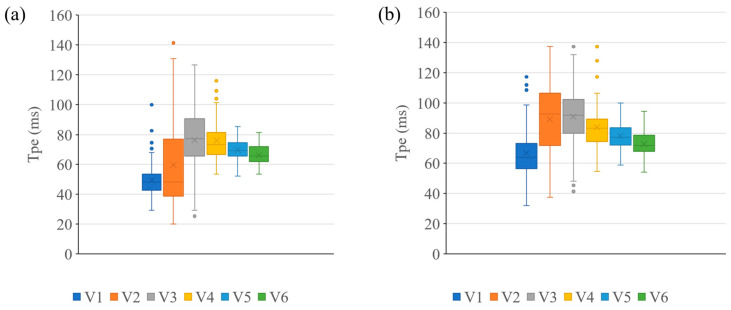
Box plots of the Tpe interval from each precordial lead. (**a**) 1st graders. (**b**) 7th graders. The shaded box shows the interquartile range, the mid-line in the box shows the median of the measured value, the whiskers show the range of the measured value, and the dots show outlier. V1 of the precordial lead is shown in light blue, V2, V3, V4, V5, and V6 are shown in orange, gray, yellow, dark blue, and green, respectively.

The evaluated ECG markers of Tpe interval, QT interval, in 1st and 7th graders and Tpe/QT ratio in 1st graders showed a normal distribution in the Shapiro–Wilk test, whereas QTc interval in 1st and 7th graders and Tpe/QT ratio in 7th graders did not (Figures S1 and S2). The comparison of these data between sexes showed that the Tpe interval, QT interval, and Tpe/QT of males and females in 7th grade, the QTc intervals of males in 1st grade and females in 7th grade were not normally distributed, while other markers showed normal distribution. The pattern of the distribution of Tpe in each precordial lead did not differ to a statistically significant extent between 1st and 7th graders. The width of the distribution of Tpe was calculated as follows: the maximum value from the V2 lead minus the minimum from either the V5 or V6 lead (Figure 2).

Table S1 shows the results of the study in the standard population. The Tpe and QTc intervals of the 7th graders were longer than those of the 1st graders and the difference in the Tpe interval between 1st graders and 7th graders was statistically significant. In contrast, the Tpe/QT ratios seemed to be almost the same. There were no significant differences in any of the indicators between boys and girls.

### 3.2. Chronological Change in Repolarization Parameters in Children with ALL

**Figure 3:**
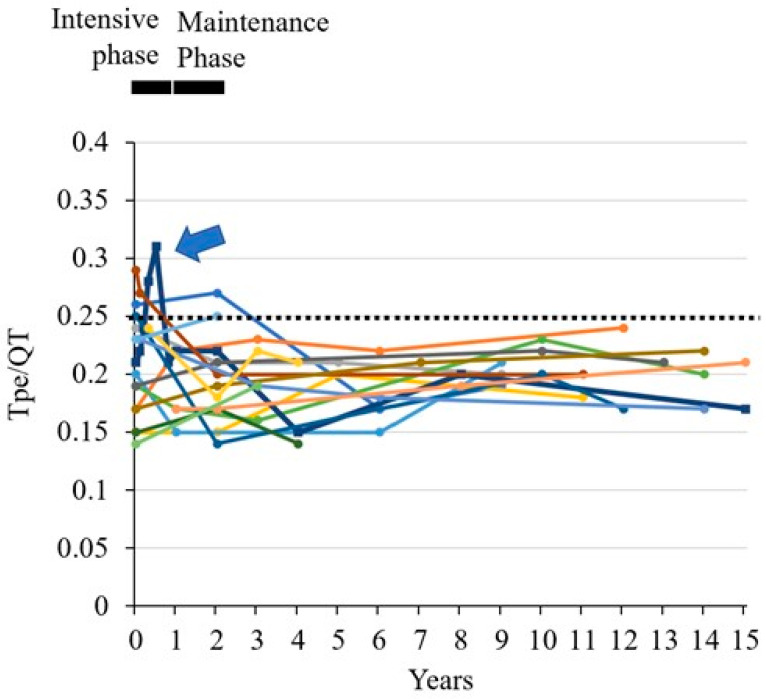
Chronological changes of the Tpe/QT ratio in 17 pediatric ALL patients. The horizontal dotted line indicates the upper level of the Tpe/QT (0.25, mean + 2SD) based on the present study. In the intensive phase of treatment for ALL, the Tpe/QT ratio of three patients treated with high-risk protocol exceeded the normal range. Among them, one patient, who is indicated by dark blue squares and a thick line (blue arrow), suffered from acute heart failure during the intensive phase of treatment for acute lymphoblastic leukemia (as shown in Figure 4). The Tpe/QT ratio of other patients converged within the normal range over a long period of time.

**Figure 4:**
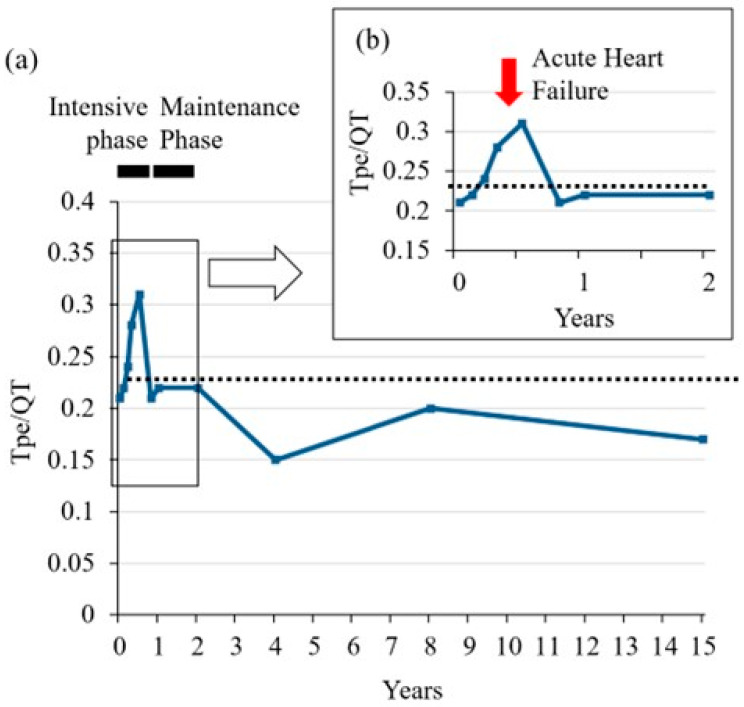
Change in the Tpe/QT ratio in a pediatric ALL patient with acute heart failure. (**a**) All follow-up terms. The horizontal dotted line indicates the upper level of Tpe/QT (0.25, mean + 2SD) based on the present study. A male patient of 2 years and 11 months of age suffered from acute heart failure (AHF) 4 months after starting treatment for ALL during the intensive phase. (**b**) First two years. The red arrow indicates the occurrence of AHF. The Tpe/QT ratio was already elevated before AHF. The high Tpe/QT ratio temporarily continued and then fell to a normal level.

The chronological data of the Tpe/QT ratios of 17 patients were evaluated. During the intensive phase of treatment, the Tpe/QT ratios from three high-risk patients exceeded the upper limit, 0.25 (mean + 2SD), whereas the QTc intervals of the same patients were within normal range (less than 450 ms) (Figure 3 and Figure S3). During long-term follow-up, the Tpe/QT ratios of all 17 patients were below the upper limit over time (Figure 3). A boy of 2 years and 11 months of age transiently demonstrated high Tpe/QT ratios under the treatment. He was treated with pirarubicin, an anthracycline (total dose, 150 mg/m^2^ [75 mg/m^2^ as doxorubicin equivalent]), prednisone, dexamethasone, vincristine, L-asparaginase, methotrexate, cytarabine, and cyclophosphamide. He suffered from bacterial infection associated with syndrome of inappropriate secretion of antidiuretic hormone. High Tpe/QT ratios were transiently detected before and under the state of acute heart failure (Figure 4), whereas the QTc intervals were within the normal range during the same period (Figure S3).

## 4. Discussion

Basic studies have shown treatment with the antitumor drug doxorubicin induced inhomogeneous prolongation of repolarization of the ventricular epicardium in mice [26]. However, clinical studies on the long-term evaluation of the VA risk have been limited especially for children. In our study, we demonstrated that the Tpe/QT ratio may be a potential marker for long-term evaluation after chemotherapy for ALL. The QTc interval was used in most previous studies on the long-term cardiotoxicity of anthracycline anti-cancer drugs. When the Tpe/QT ratio is applied as an age-independent indicator for VR, it would be preferable for the evaluation of the long-term risk of VA from childhood to adulthood. For this purpose, the Tpe/QT ratio was almost constant at 0.21 ± 0.02, and the Tpe/QT ratio would be a practical candidate for monitoring the long-term risk of VA during childhood and adulthood.

In this study, one pediatric patient exhibited a transient Tpe/QT ratio substantially above the normal range, despite their QTc remaining within normal limits during the period of complications. Additionally, two other patients demonstrated transient elevations throughout the initial treatment phase. These findings may be attributed to the presence of temporary myocardial injury. While none of the three patients displayed re-elevations during subsequent long-term observations, they warrant heightened vigilance for potential arrhythmias in the future. In the current investigation, no patient exceeded the reference values for the chronic phase during an observation period of up to 15 years. Nonetheless, considering the lifelong management required for childhood cancer survivors, it is imperative to conduct periodic follow-up evaluations over several decades. Future investigations focused on long-term management outcomes will serve to illuminate the relevance and importance of this specific index of Tpe/QT ratio.

From a histological point of view, three types of electrophysiologically distinctive cells have been identified in the ventricular myocardium: endocardial, epicardial, and M cells. Differences in the time course of repolarization of these three ventricular myocardial cell types prominently contribute to the inscription of the electrocardiographic T-wave [27]. Previously, the Tpe interval was taken to be a reflection of the transmural dispersion of repolarization (TDR) [28]. However, in recent reports, Tpe does not correlate with transmural dispersion of repolarization but is an index of total dispersion of repolarization [29,30].

There are few reports on the normal value of the Tpe interval in children. According to a report by Brash et al., in Italy, the Tpe interval was 76 ± 9 ms at 6–8 years of age and 86 ± 9 ms at 12–14 years of age, which was 6 ms and 8 ms longer than the values in our study, respectively (Table 2) [19]. We speculate that this difference may be derived from—at least in part—the method used to calculate the Tpe interval; they selected the longest Tpe interval from all precordial leads, whereas we only used the V5 lead for the measurement of the Tpe interval. As shown in Figure 2, the Tpe interval in V2 measurements had the largest deviation due to difficulty in deciphering the peak and end of the T-wave.

In our study in Japan, the mean Tpe intervals at 6–7 and 12–13 years of age were 70 ms and 78 ms, respectively, and Takenaka et al. reported that the average of the Tpe interval at 15–45 years of age was 86 ms (Table 2) [31]. Hence, the Tpe interval seems to increase with age from childhood to adulthood, which was also observed in the Italian study (Table 2) [19]. In contrast, in our study, the mean ± SD Tpe/QT ratios in 1st and 7th graders were 0.21 ± 0.02 ms and 0.22 ± 0.02 ms, respectively, while that in the previous Italian study was 0.21 ± 0.02 ms in children and adults (Table 2), suggesting that the Tpe/QT ratio was consistent, irrespective of age difference or ethnicity [19]. Gupta et al. reported that the Tpe/QT ratio was almost constant, despite dynamic physiological changes in the heart rate. Consequently, the Tpe/QT ratio may have a substantial advantage in pediatric and adult studies for predicting VA over the long term.

The measurement method is a major point to be improved for the clinical use of the Tpe/QT ratio (i.e., the Tpe interval and Tpe-d were manually measured). Currently, manual measurement is commonly employed, which presents several limitations such as subjectivity, inter-observer variability, and measurement errors. Automated measurement methods may offer advantages, but their accuracy and consistency compared to manual measurement methods remain a matter of debate [32]. For instance, accurate identification of the peak and end of the T-wave can be challenging if the T-wave forms a complex type. Thus, further investigations comparing the pros and cons of automated versus manual measurements are warranted to establish the clinical relevance of these indices. In the recent report, a more thorough evaluation would have also included quantification and investigation of likely more diagnostically and prognostically powerful advanced measures from stored digital ECG recordings, such as quantification of T-wave morphology/complexity [33]. Another limitation of this study was its single-center, retrospective design and the small sample size of pediatric patients with ALL; additionally, it might introduce selection bias. From the perspective of generalizability, our patients were treated at a single center, but the protocol we implemented is common across all JACLS facilities, and our facility also followed it. Moreover, the event-free survival rate was approximately 87%, which is comparable to the overall result, suggesting that it was a typical patient group [34]. The median follow-up period for our patients was 11 years, which was longer than the period reported by the JACLS as a whole (6.6 years) [1]. On the other hand, considering that there were 1138 non-T cell ALL patients treated under the JACLS ALL02 protocol, and we analyzed only 17 of them, it might limit the ability to establish causality. Therefore, future multicenter studies with larger sample sizes are warranted to validate our findings. Additionally, it is necessary to test these indices in other pediatric hematological malignancies and solid tumors that are treated with anthracyclines.

## 5. Conclusions

We evaluated the ECG markers for VR in childhood lymphoblastic leukemia (ALL), from childhood to adulthood. For this purpose, we establish the reference values of the Tpe interval and Tpe/QT ratio in healthy Japanese children. When the Tpe/QT ratio is applied as an age-independent indicator for VR, it would be preferable for the evaluation of the long-term risk of VA. This method might help the early detection of cardiac complications, especially arrhythmia, such as anthracycline toxicity.

## Figures and Tables

**Table 1:** Clinical characteristics of the enrolled children with ALL.

Total Subjects (Number)	17
Male/Female (number)	11/6
Age at onset (years) [Median]	1~11 [4]
Classification by risk criteria (number)	
Standard-Risk [Relapse]	5 [1]
High-Risk	9
Extremely-High-Risk [Relapse]	2 [1]
Philadelphia chromosome-positive	1
Follow-up period (years) [Median]	2–15 [11]

**Table 2:** Comparison of the current and previous reports.

Nationality	Japanese (Oita City)		Japanese (Kyoto City)	Italian		
Age (Years)	6–7	12–13	15–45	6–8	12–14	20–94
Number of subjects	100	100	33	<200	<200	202
	Mean ± SD		Mean	Mean ± SD		
Tpe (ms)	70 ± 7	78 ± 9	86	76 ± 9	86 ± 9	87 ± 9.5
QT (ms)	328 ± 22	364 ± 27	358	330 ± 19	356 ± 21	373 ± 26
QTc (ms)	365 ± 20	385 ± 20	402	399 ± 26	402 ± 21	407 ± 20
Tpe/QT	0.21 ± 0.02	0.22 ± 0.02	NE	0.21 ± 0.02	0.21 ± 0.02	0.21 ± 0.02

SD, standard deviation; NE, not examined.

## Data Availability

All data, materials, and codes used in this study are available upon request from the corresponding author.

## Supplementary Materials

The following supporting information can be downloaded at: https://www.mdpi.com/article/10.3390/jcm12144723/s1, Figure S1: Distribution of the ventricular repolarization marker values in 1st graders; Figure S2: Distribution of ventricular repolarization marker values in 7th graders; Figure S3: Chronological changes of ventricular repolarization marker values in 17 pediatric ALL patients; Table S1: Ventricular repolarization markers of Japanese school children.

## Abbreviations

ALL	acute lymphoblastic leukemia
BS	Brugada syndrome
ECG	electrocardiography
LQTS	long QT syndrome
QTc	correct QT
QTd	QT dispersion
Tpe	Tpeak-end
VA	ventricular arrhythmia
VR	ventricular repolarization
