# Supplementary material for: Predicting Long-Term Ventricular Arrhythmia Risk in Children with Acute Lymphoblastic Leukemia Using Normal Values of Ventricular Repolarization Markers Established from Japanese Cohort Study
Source: J Clin Med. 2023 Jul 17;12(14):4723. doi: 10.3390/jcm12144723 (PMC10381239; doi:10.3390/jcm12144723)
Supplement: Supplementary file 1 [file jcm-12-04723-s001.zip › jcm-2475431-supplementary.pdf]

TABLE S1 Ventricular repolarization markers of Japanese school children

Age	1st graders (6-7 years old)		7th graders (12-13 years old)	
Subjects	100 (49/51)		100 (56/44)	
	Mean	SD	Mean	SD
Heart rate (bpm)	82	11	72	13
Tpe (ms)	70 (69/70)	7 (7/7)	78 (77/80)	9 (8/9)
QT (ms)	328 (323/329)	22 (20/24)	364 (362/365)	27 (27/27)
QTc (ms)	365 (364/366)	20 (18/21)	385 (384/386)	20 (20/20)
Tpe/QT	0.21 (0.21/0.21)	0.02 (0.02/0.02)	0.22 (0.21/0.22)	0.02 (0.02/0.02)

Values in parentheses are the data of boys/girls. SD, standard deviation

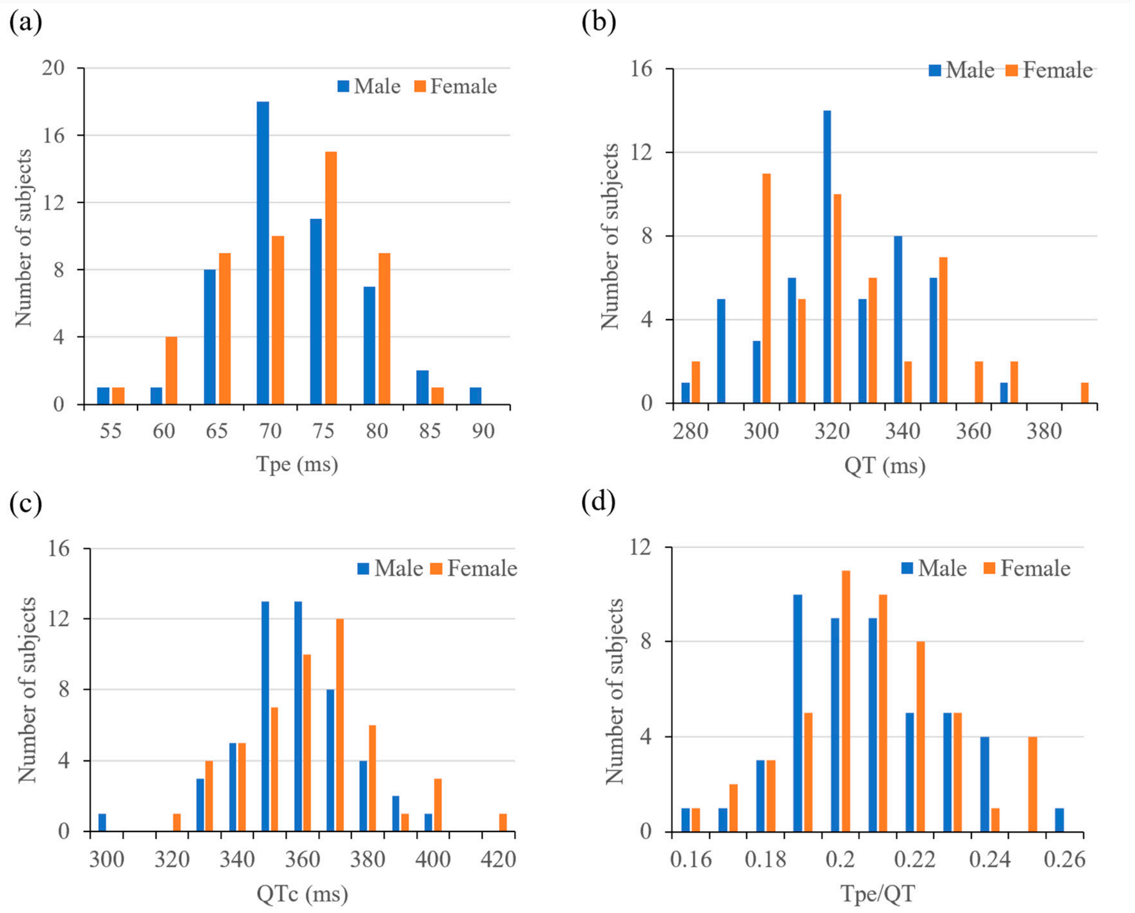

Figure S1. Distribution of the ventricular repolarization marker values in 1st graders.

The (A) Tpe interval, (B) QT interval, (C) QTc interval, and (D) Tpe/QT ratio. The values of Tpe interval, QT interval, and Tpe/QT ratio were found to be normally distributed by the Shapiro-Wilk test (P-value 0.133, 0.166, and 0.183, respectively), whereas the QTc interval was not normally distributed (P-value 0.014). Among them, the Tpe interval, QT interval, -and Tpe/QT ratio values in males and females were normally distributed (P-value male/female=0.084/0.311, 0.066/0.071,and 0.135/0.316, respectively). The QTc

interval of females were normally distributed (P-value 0.065), whereas the QTc interval of males was not normally distributed (P-value 0.005).

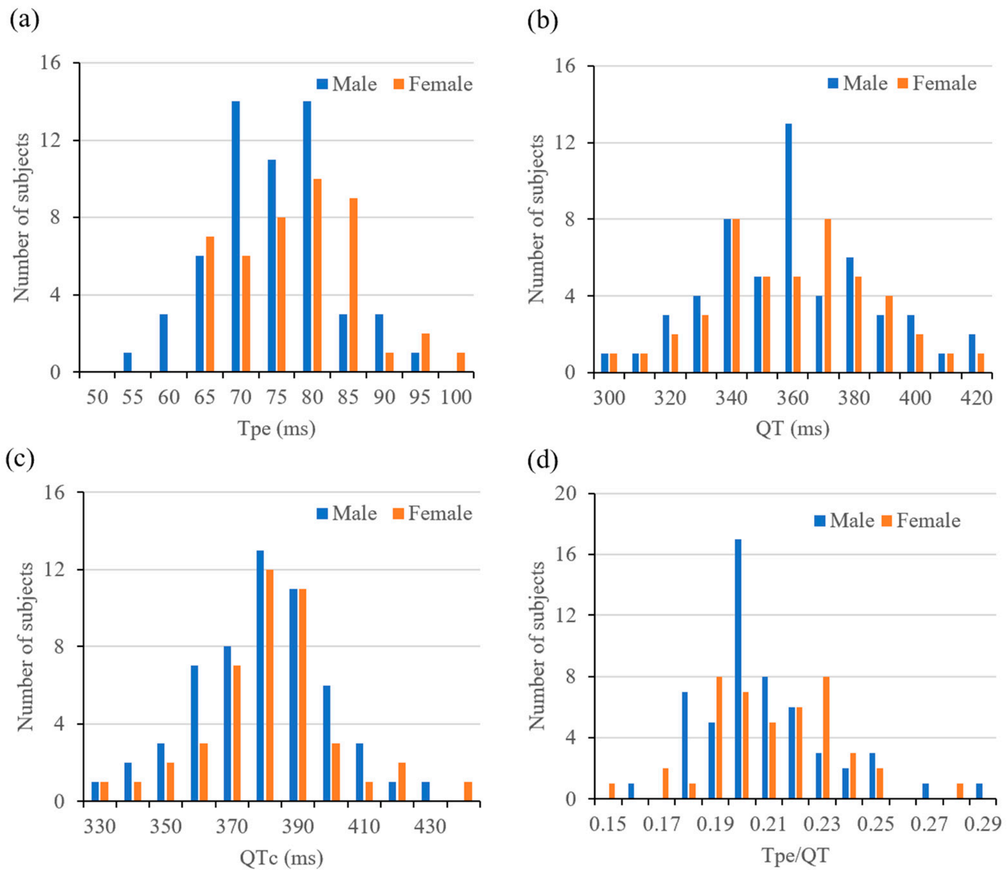

Figure S2. Distribution of ventricular repolarization marker values in 7th graders.

(A) Tpe interval, (B) QT interval, (C) QTc interval, and (D) Tpe/QT ratio. The Tpe intervaland QT intervalwere found to be normally distributed by the Shapiro-Wilk test (P-value 0.060 and 0.157, respectively), whereas the QTc interval and Tpe/QT ratio were not normally distributed (P-value 0.02 and 0.001The Tpe interval, QT interval and Tpe/QT of males and females were not normally distributed (P-value male/female=0.01/0.03, 0.018/0.042, 0.002/0.012, respectively). QTc interval of males were normally distributed (P 0.108), whereas the QTc intervals of females were not normally distributed (P 0.004).

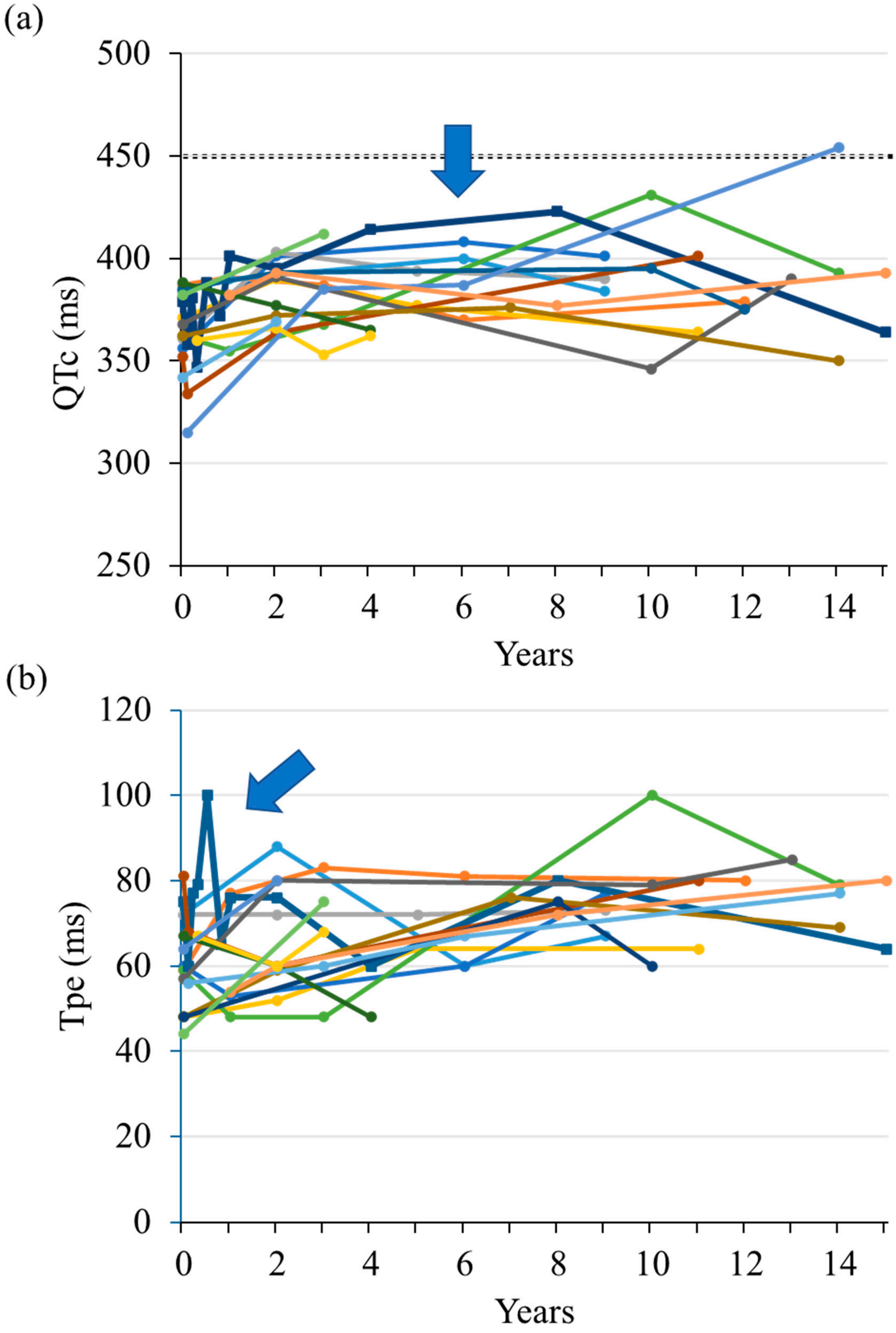
(a)
500
450
400
350
300
250
QTc (ms)
0
2
4
6
8
10
12
14
Years
(b)
120
100
80
60
40
20
0
Tpe (ms)
0
2
4
6
8
10
12
14
Years

Figure S3. Chronological changes of ventricular repolarization marker values in 17 pediatric ALL patients.

(A) QTc interval, (B) Tpe interval. The horizontal dotted line indicates the upper level of QTc (450 ms) based on the guideline. [1] The QTc interval in all patients was less than 450 milliseconds during the intensive phase of treatment for ALL. A patient, who is indicated by the blue arrow with dark blue squares with a thick line, suffered from acute heart failure during the intensive phase of treatment of ALL. Transient prolongation of the Tpe interval was observed in the patient during the same period; however, the QTc interval remained in the normal range during treatment and throughout the follow-up period.
